# The complete chloroplast genome sequence of *Chrysophyllum cainito*, a semidomesticated species

**DOI:** 10.1080/23802359.2020.1768964

**Published:** 2020-05-27

**Authors:** Cheng Zheng, Zi-Yan Liu, Jin Liu

**Affiliations:** Yunnan Institute of Tropical Crops, Xishuangbanna, China

**Keywords:** *Chrysophyllum cainito*, chloroplast genome, sequencing

## Abstract

*Chrysophyllum cainito* is a semi-domesticated species widely cultivated in tropical regions, such as the Americas and Southeast Asia. In Yunnan, Guangdong, and Fujian Provinces, China, *C. cainito* is planted as an edible tropical fruit that was introduced from Southeast Asia. In this study, the chloroplast genome sequence of *C. cainito* was assembled and characterized using Illumina sequencing. The whole chloroplast genome of *C. cainito* is 158,841 bp long and consists of four regions: a large single-copy region (LSC, 88,256 bp), two inverted repeat regions (IRs, 25,958 bp), and a small single copy (SSC, 18,669 bp) region. The composition of the four bases in the circular chloroplast genome is 31.20% A, 32.00% T, 18.02% G, and 18.78% C, and the GC content of the entire *C. cainito* chloroplast genome is 36.8%. A total of 129 genes were annotated in the *C. cainito* chloroplast genome, of which 84 were protein-coding genes, 37 were transfer RNA (tRNA) genes, and eight were ribosomal RNA (rRNA) genes. The phylogenetic analysis indicated that *C. cainito* was most closely related to *Pouteria campechiana.* This study provides a foundation for further investigation of chloroplast genome evolution and genetic variation within semi-domesticated species.

*Chrysophyllum cainito* L. (Sapotaceae), commonly known as caimito or star apple, is a neotropical tree valued for its ornamental quality and its edible fruit (Morton 1987). The species is native to the Caribbean and the West Indies and is primarily distributed in tropical regions such as the Americas and Southeast Asia. *Chrysophyllum cainito* has been introduced from Southeast Asia to Yunnan, Guangdong, and Fujian Provinces, China, as tropical edible fruit. Some observed differences between cultivated and wild individuals suggest that the cultivated cainito trees are semi-domesticated (Parker et al. 2010); therefore, it is an excellent system to study anthropogenic impacts on the distribution of a neotropical fruit tree (Petersen et al. 2014). Understanding the patterns and processes associated with domestication has implications for crop development and agricultural biodiversity conservation (Petersen et al. 2012). Chloroplast DNA contains a wealth of genetic information, which can provide useful molecular markers for future genetic studies (Liu et al. 2018). In this manuscript, we characterized the complete chloroplast genome sequence of *C. cainito* as a resource to study anthropogenic impacts on genetic and phenotypic variation within cultivated species.

Fresh leaf samples of *C. cainito* were collected from Xishuangbanna Tropical Flowers and Plants Garden (22°01′8.64″N 100°47′20.07″E), south Yunnan, China, and frozen with liquid nitrogen. Genomic DNA was isolated using the Dneasy Plant Mini Kit (Qiagen) and stored in the ultra-low temperature specimen library at YITC (specimen accession number: YITC-2019-FZ-C-006). The isolated DNA was sent to BGI Shenzhen for library construction and genome sequencing on the Illumina Hiseq 2000 Platform (Illumina, San Diego, CA). A total of 6.0 Gbp reads in fastq format were obtained and used in the chloroplast genome assembly. The complete chloroplast genome was annotated with Dual Organelle GenoMe Annotator (DOGMA; Wyman et al. 2004) and submitted to GenBank (http://www.ncbi.nlm.nih.gov/) under the accession number MT435527. A physical map of the chloroplast genome was generated by OGDRAW (http://ogdraw.mpimp-golm.mpg.de/) (Lohse et al. 2013).

The whole chloroplast genome of *C. cainito* is 158,841 bp and consists of four regions: a large single-copy region (LSC, 88,256 bp), two inverted repeat regions (IRs, 25,958 bp), and a small single copy (SSC, 18,669 bp) region. The composition of the four bases in the circular chloroplast genome is 31.20% A, 32.00% T, 18.02% G, and 18.78% C, and the GC content is 36.8%. A total of 129 genes were annotated in the *C. cainito* chloroplast genome, of which 84 were protein-coding genes, 37 were transfer RNA (tRNA) genes, and eight were ribosomal RNA (rRNA) genes. The protein-coding genes are involved in the following biological functions: photosystem I, photosystem II, cytochrome b/f complex, ATP synthase, NADH dehydrogenase, RNA polymerase, clpP, infA, matK, and hypothetical chloroplast reading frames (ycf).

In order to verify the evolutionary relationship of *C. cainito*, we constructed a maximum likelihood tree based on the complete chloroplast genome sequences from 15 published plant species. MAFFT (Katoh and Standley 2013) was used for multiple sequence alignment and MEGA7.0 (Kumar et al. 2016) was used for maximum-likelihood (ML) analysis (Figure 1). Of the included chloroplast genomes, the results indicated that *C. cainito* was most closely related to *Pouteria campechiana*. This study provides a foundation for further investigation of chloroplast genome evolution and genetic variation within semi-domesticated species.

**Figure 1.**
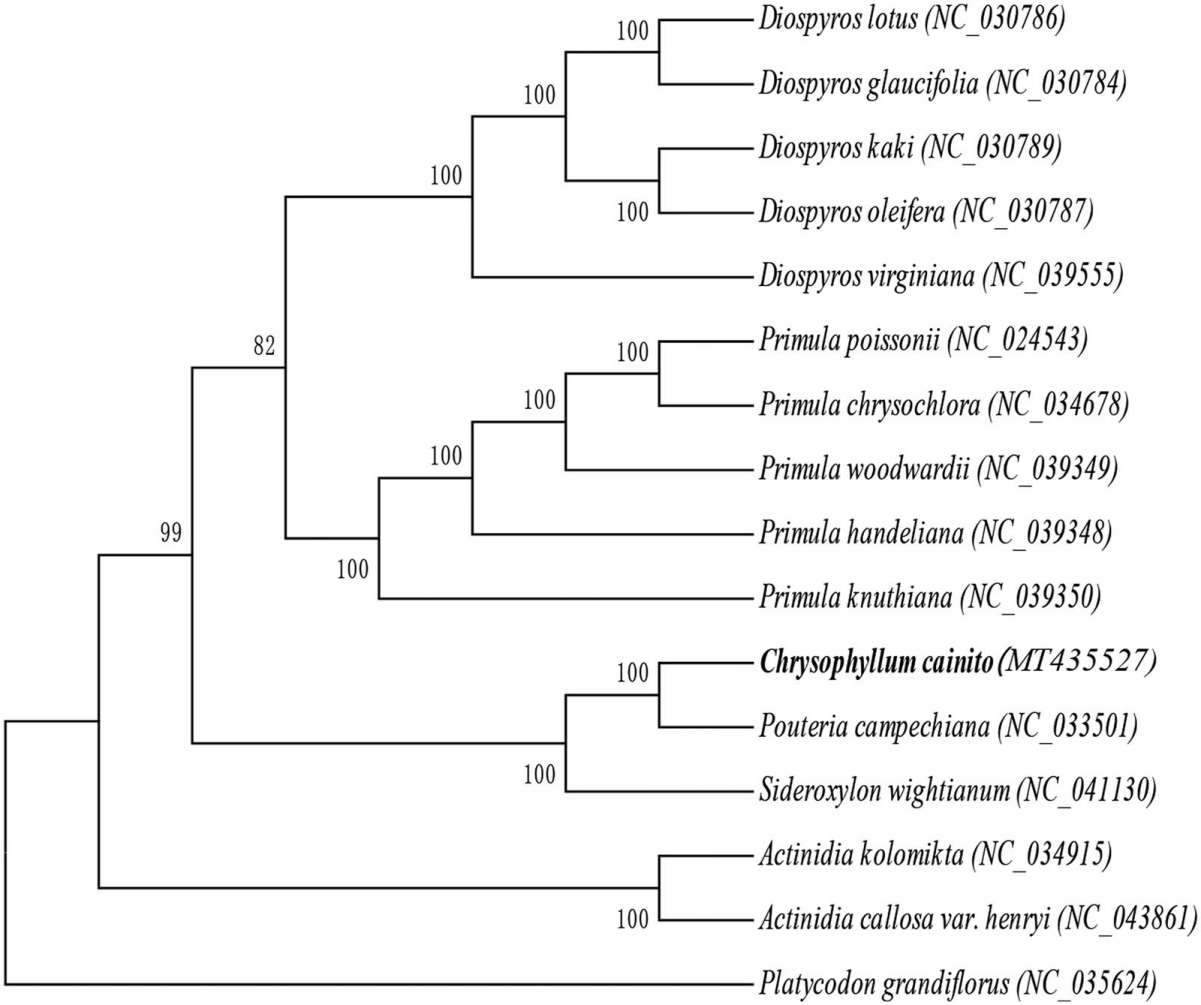
Maximum-likelihood phylogenetic tree of *C. cainito* and 15 other species (five species of the Ebenaceae family, five species of the Primulaceae family, two species of the Sapotaceae family, two species of the Actinidiaceae family, and *Platycodon grandiflorus*, which belongs to the Campanulaceae family and was used as the outgroup). The bootstrap value was set to 1000. The species and chloroplast genome accession numbers for tree construction are: *C. cainito* (MT435527)*, Diospyros glaucifolia* (NC_030784), *Diospyros lotus* (NC_030786), *Diospyros oleifera* (NC_030787), *Diospyros kaki* (NC_030789), *Diospyros virginiana* (NC_039555), *Primula poissonii* (NC_024543), *Primula chrysochlora* (NC_034678), *Primula handeliana* (NC_039348), *Primula woodwardii* (NC_039349), *Primula knuthiana* (NC_039350), *Pouteria campechiana* (NC_033501), *Sideroxylon wightianum* (NC_041130), *Actinidia kolomikta* (NC_034915), *Actinidia callosa var. henryi* (NC_043861), and *P. grandiflorus* (NC_035624).

## Data Availability

The data that support the findings of this study are openly available in GenBank at https://www.ncbi.nlm.nih.gov/, reference number MT435527.
